# 

**Published:** 1903-11-07

**Authors:** 


**Publications.**

### CHARTS.

PRICES (Post or Carriage Paid):—

1,000, 25s.; 500, 13s. 6d.; 250, 7s. 6d.; 100, 3s. 6d.; 50, 2s.; 25, 1s. 3d.

SPECIMENS POST FREE.

THE BEST AND CHEAPEST PUBLISHED.

GOULD'S TEMPERATURE CHARTS, Morning and Evening.

GOULD'S FOUR-HOUR CHARTS, Printed in Violet for hours 2, 6, 10.

GOULD'S FOUR-HOUR CHARTS, Printed in Chocolate for hours 4, 8, 12.

GOULD'S TWO-HOUR CHARTS.

BENTON'S DIET CHARTS, each Chart lasting two days.

BOARDS to hold charts when in use, 9d. each, 8s. 6d. per dozen.

PRIVATE NURSING CHARTS, lasting 48 hours.

CHARTS for taking the Temperature of Sick Rooms.

USED IN ALMOST ALL THE PRINCIPAL HOSPITALS AND INFIRMARIES IN THE UNITED KINGDOM.

**WODDERSPOON & CO., 6 Gate Street, Lincoln's Inn Fields, W.C.**

---

WORKS BY Mr. McADAM ECCLES, M.S., F.R.C.S.,
Assistant Surgeon St. Bartholomew's Hospital, &c.

### HERNIA.

SECOND EDITION, 1902. Price 7/6 net. Illustrated by Numerous Photographs.

### THE IMPERFECTLY DESCENDED TESTIS.

Just published. Profusely Illustrated. Price 7/6.
London: BAILLIÈRE, TINDALL & COX.

---

2nd Edition. Revised and Enlarged. Now Ready.
Post 8vo. pp. xv.-268, and 39 Illustrations in the text.
Cash price 4/6, postage 4d.

### PRACTICAL TEXT-BOOK OF MIDWIFERY FOR NURSES.

By ROBERT JARDINE, M.D. Edin., M.R.C.S. Eng., F.F.P. and S. Glas.
Professor of Midwifery in St. Mungo's College, Glasgow; Physician to the Glasgow Maternity Hospital; late President of the Glasgow Obstetrical and Gynæcological Society.

"It is the best suited for its purpose of any book of the kind which we have come across. . . . For nurses the little book is an improvement on any similar book of the kind with which we are familiar."
*The Dublin Journal of Medical Science.*

W. F. CLAY, 18 Teviot Place, Edinburgh.

---

### THE HANDBOOK OF THE OPEN-AIR TREATMENT

By Dr. CHARLES REINHARDT
(Visiting Physician to Hailey Sanatorium, Wallingford, and Stourfield Park Sanatorium, Bournemouth),
Contains Views, Descriptions, and Information respecting 30 Sanatoria in Great Britain.
Price: Cloth, 2/6; Paper, 1/- (postage 4d.)
The "HEALTH RESORT" Publishing Office, 379 Strand, London, W.C.

---

Crown 8vo. cloth, price 5s. net; by post, 5s. 4d.
*Second Edition of*
DR. HEYWOOD SMITH'S

### PRACTICAL GYNÆCOLOGY.

This is a genuine handbook on the subject, intended for ready reference by busy practitioners.
H. J. GLAISHER, 57 Wigmore Street, Cavendish Square, W.

---

Crown 8vo. Illustrated. Price 2s. 6d. net.

### ANÆSTHETICS. By J. BLUMFELD,

M.D.Cantab., Senior Anæsthetist to St. George's Hospital; Anæsthetist to the Grosvenor Hospital for Women and Children, London, and to the National Dental and Metropolitan Hospitals.

*Lancet.*—"....In writing this handbook the author has been quite successful." *Australasian Medical Gazette.*—"We can strongly commend this book." *Medical Chronicle.*—"The book is one which we can heartily recommend to senior students and practitioners."

London: BAILLIÈRE, TINDALL & COX, 8 Henrietta St., Covent Garden.

---

JUST ISSUED. EIGHTH EDITION. Crown 8vo. 10s. 6d.

### PHARMACY, MATERIA MEDICA AND THERAPEUTICS

Is now rewritten and printed in large type, and is made a companion to the following volume. It contains an account of the action **of every drug in the B.P.**, as well as a section upon all the newest drugs, with an Introduction to the Art of Dispensing. With woodcuts, prescriptions, &c.

Also FOURTH EDITION. 1055 pages, 8vo. 16s.

### DICTIONARY OF TREATMENT.

Containing a Revised and up-to-date Article upon every Diseased Condition, whether Medical, Surgical, Gynæcological, or Ophthalmological, as well as Articles upon Poisoning, Drowning, and the various Surgical Emergencies and Accidents, arranged for Rapid Reference.

By W. WHITLA, M.A., M.D., Professor of Materia Medica, Queen's College, Belfast; Senior Physician to the Royal Victoria Hospital, &c.

The Publisher will supply both volumes, carriage paid, for 21/-

London: HENRY RENSHAW, 356 Strand.

---

Crown 8vo., price 3/6.
With a "Plea for the State Registration of Nurses."

### MOCK NURSES OF THE LATEST FASHION.

By FREDERICK J. GANT, F.R.C.S.,
Consulting Surgeon to the Royal Free Hospital.

BAILLIÈRE, TINDALL & COX,
Henrietta Street, Covent Garden, London.

---

Second Edition.

### CLINICAL LECTURES ON NEURASTHENIA.

By THOMAS D. SAVILL, M.D.Lond.

"Deal very exhaustively with the subject, and will be read with great interest."—*The Lancet.*
"It should be in the hands of every practitioner who wishes to keep himself well informed on this important subject."—*Treatment.*

H. J. GLAISHER, 57 Wigmore Street, W. 5/- net; 5/4 by post.

NOTICE TO CORRESPONDENTS.

All MSS., Letters, Books for Review, and other matters intended for the Editor should be addressed THE EDITOR, "THE HOSPITAL," THE HOSPITAL BUILDINGS, 28 & 29 SOUTHAMPTON STREET, STRAND, W.C.

The Editor cannot undertake to return rejected MS., even when accompanied by stamped directed envelope.

**Notice to Advertisers and Subscribers.**

All Advertisements, Orders for Copies of THE HOSPITAL, and Business Communications should be addressed to **THE MANAGER (Not to the Editor),** THE HOSPITAL, 28 & 29 Southampton Street, Strand, London, W.C.

The Cost of Subscription, post free, is as follows:—Per week, 3½d.; per quarter, 3s. 9d.; per half-year, 7s. 6d.; per annum, 15s. Foreign and Colonial:—Per annum, 19s. 6d., payable, in all cases, in advance.

**NOTICE.—Vol. XXXIV. of "The Hospital" (April 1903 to September 1903), in handsome cloth binding, will shortly be on sale at the office of this Journal, price 7s. 6d. Cases for binding the Numbers contained in this Volume 1s. 6d. Index to Vol. XXXIV., 2½d., post free.**

**The Monthly part for November is now ready, Price 1s., by post, 1s. 3d.**

### THE STUDENT OF MEDICINE.

### APPOINTMENTS.

**Charles M. Anderson**, M.D., C.M.Edin., M.R.C.S.E., L.R.C.P.Lond., Clinical Assistant to the Samaritan Free Hospital. **G. P. Bletchley**, M.B.Lond., M.R.C.S., Certifying Factory Surgeon for the Nailsworth District, Gloucestershire. **G. H. Bowlby**, M.D., L.R.C.P., M.R.C.S., as Clinical Assistant to the Chelsea Hospital for Women. **S. Bridger**, M.R.C.S., L.R.C.P.Lond., Certifying Factory Surgeon for the Southend District, Essex. **Oswald A. Browne**, M.D Cantab., F.R.C.P. Lond., Consulting Physician to the Metropolitan Hospital. **Edmund Cautley**, M.D.Cantab., F.R.C.P.Lond., Pysician to the Metropolitan Hospital. **J. Craig**, M.B., Certifying Factory Surgeon to the Biddulph District, Staffordshire. **G. W. Bampfylde Daniell**, M.R.C.S., L.R.C.P., Instructor in Anæsthetics to the Royal Infirmary, Edinburgh. **Geo. H. Edington**, M.D., M.R.C.S.Eng., L.R.C.P.Lond., Lecturer in Surgery at Western Medical School, Glasgow. **F. Clayton Gould**, L.R.C.P.Edin., L.R.C.S.Edin., L.F.P.&S.Glasg., Medical Referee to the Star Life Assurance Company, Birmingham District. **Arthur Francis Hamilton**, M.B.Lond., M.R.C.S. L.R.C.P., appointed House Surgeon to the West London Hospital. **F. C. Hanson**, L.R.C.P.&S.Irel., Certifying Factory Surgeon for the Bray District, Wicklow. **F. H. Jacob**, M.D.Lond., M.R.C.P., Assistant Physician to the Nottingham Children's Hospital. **H. Percy Job**, M.R.C.S., L.R.C.P.Lond., Medical Officer for the Coddington District of the Newark Union. **Thomas Massie**, M.B., C.M.Aberd., Medical Officer at the St. George's Workhouse, Southwark Union, London, S.E. **J. Lewis Owen**, M.R.C.S. L.R.C.P.Lond., Medical Officer of Health for the Holyhead Rural District. **W. R. Penny**, M.R.C.S., L.R.C.P., Certifying Factory Surgeon for the Greenodd District, Lancashire. **Mrs. Agnes F. Savill**, M.D.Glasg., Clinical Assistant to the New Hospital for Women, Euston Road. **Harold Scurfield**, M.D.Edin., D.P.H.Camb., Medical Officer of Health for Sheffield. **C. P. Stewart**, M.B., C.M.Edin., Certifying Factory Surgeon for the Perth District, Perthshire. **E. Stork**, M.B.Lond., Medical Officer of Health for Bury St. Edmunds. **Edwin R. Thomas, junior**, L.R.C.P.&S.Edin., L.F.P.S.Glasg., Medical Officer of Health to the Beaumaris Town Council. **Hugh Thursfield**, M.D.Oxon., M.R.C.P.Lond., Assistant Physician to the Metropolitan Hospital. **George Albert Turner**, M.B., B.S.Aberd., D.P.H., Medical Officer of Health for Kimberley. **David Waterston**, M.A., M.D., F.R.C.S.E., F.R.S.E., Senior Assistant in Anatomy and Lecturer in Regional Anatomy in Edinburgh University.

### "The Hospital" Institutional Library.

"Hospitals and Asylums of the World." 4 Vols., with a Portfolio of Plans, complete £12 12s.

"Burdett's Hospitals and Charities, 1903." 5s. net.

"Burdett's Official Nursing Directory, 1903." 3s. net.

"Cottage Hospitals, General, Fever, and Convalescent." 10s. 6d.

"Uniform System of Accounts for Hospitals and Public Institutions." New Edition. 4s. net.

"Hospital Expenditure: The Commissariat." 2s. 6d.

"A Legal Handbook for the Use of Hospital Authorities." 2s. 6d.

"The Cottage Hospital Case Book or Register of Patients." 10s. and 12s. 6d.

All these are published by the SCIENTIFIC PRESS, Ltd., and may be obtained through any bookseller, or direct from the publishers, 28 and 29 Southampton Street, Strand, London, W.C.

### THE PRIVATE NURSE'S OWN NOTE-BOOK.

By SISTER EVA (with photo of Authoress). Price 6d. post free from BEMROSE & SONS, Derby.

An exceedingly well got up and useful little volume intended to fill a long felt want, containing tear-off pages for expenses incurred at cases, receipts for same, notes and valuable hints on private nursing, dainty dishes for the sick. Also spare pages for Nurse's notes.

### A NEW CASE BOOK FOR PRIVATE NURSING.

### To Nurses.

Dear Nurses,—We have learnt by observation that something larger and simpler is needed in the nursing of private cases than can be found amongst the ordinary case books published. Therefore most nurses buy an exercise book, and arrange each one themselves. Now you need not waste your valuable time in ruling and writing. Our Case Book is ready for use for the price of an exercise book. It is much neater and clearer than anything you can arrange for yourselves. If you like our book, please show it to others. They also will be glad to render their reports in concise form. It helps the doctor, and is appreciated by him.

Yours faithfully,
The Publishers.

Arranged for Day and Night Nurse's Report, with rulings in accordance with what has been found in practice to be the most suitable and approved System for Pay Hospitals, Nursing Homes, Private Nurses, &c.

DAY NURSE'S REPORT.

*Date............*

| Time | Nourishment | Quantity | Urine | Stimulants, Medicines, and Remarks |
|---|---|---|---|---|
| | | | | |

| Total Nourishment | Bowels | Summary |
|---|---|---|
| | | |

(Facsimile of ruling.)

Price 3d. net. Post free 4½d.

The Size of an Exercise Book (7½ in. by 9¼ in.).

*Obtainable of all Booksellers.*

Price 3d. net. Post free 4½d.

*Special Quotations for a quantity can be procured from the Publishers,*
THE SCIENTIFIC PRESS, LIMITED, 28 & 29 Southampton Street, Strand, London, W.C.

THE

### BURDETT SERIES

OF

### POPULAR TEXT-BOOKS on NURSING.

**"Valuable little handbook."**

*Nursing Notes.*

THE BURDETT SERIES.

**"Can be strongly recommended to nurses."**

*Treatment.*

Small Crown 8vo. Bound in cloth boards. Price **1/-** each post free; or the Series complete **10/-** post free.

**Each Volume contains about 100 pages written by a competent authority on the subject treated.**

No. 1.—**PRACTICAL HINTS ON DISTRICT NURSING.** By Amy Hughes.

No. 2.—**HOSPITAL AND PRIVATE NURSING.** By S. E. Orme.

No. 3.—**THE MIDWIVES' POCKET-BOOK. With Prefatory Chapter on the Midwives' Bill.** By Honnor Morten, Author of "The Nurse's Dictionary," &c., &c.

No. 4.—**FEVERS AND INFECTIOUS DISEASES:** their Nursing and Practical Management. By William Harding, M.D.Ed., M.R.C.P.Lond., Author of "Mental Nursing," &c.

No. 5.—**NOTES ON PHARMACY AND DISPENSING FOR NURSES.** By C. J. S. Thompson.

No. 7.—**THE CARE OF CONSUMPTIVES.** By W. H. Daw, M.R.C.S., L.R.C.P.Lond.

No. 8.—**HOME NURSING OF SICK CHILDREN.** By J. D. E. Mortimer, M.B., F.R.C.S., L.S.A.

No. 9.—**MENTAL NURSING.** By William Harding, M.D.Ed., M.R.C.P.Lond.

No. 10.—**SICK NURSING AT HOME.** By Miss L. G. Moberly.

No. 11.—**GYNÆCOLOGICAL NURSING.** By G. A. Hawkins-Ambler, F.R.C.S., Author of "The Commonwealth of the Body."

No. 12.—**SYLLABUS OF LECTURES TO NURSES.** By Andrew Davidson, M.D.

*To be obtained of all Booksellers, or of*

The SCIENTIFIC PRESS, LTD., 28 & 29 Southampton Street, Strand, London, W.C.

Standard Works **J. & A. CHURCHILL'S** Latest Editions

### Books for Nurses.

### NURSING.

**Nursing, General, Medical and Surgical, with an Appendix on Sick Room Cookery.** By WILFRED J. HADLEY, M.D., F.R.C.P., F.R.C.S., Physician and Pathologist to the London Hospital, Lecturer to Nurses at the London Hospital Nursing School . . . . 3s. 6d.

### MEDICINE.

**Lectures on Medicine to Nurses.** By HERBERT E. CUFF, M.D., F.R.C.S., Medical Superintendent, North Eastern Fever Hospital, London. Fourth Edition. With 29 Illustrations . . . . . 3s. 6d.

### INFANT FEEDING.

**The Natural and Artificial Methods of Feeding Infants and Young Children.** By EDMUND CAUTLEY, M.D., F.R.C.P., Physician to the Belgrave Hospital for Children; late Casualty Physician and Assistant Demonstrator of Physiology, St. Bartholomew's Hospital. Second Edition. 7s. 6d.

### DIET.

**Diet and Food considered in relation to Strength and Power of Endurance, Training and Athletics.** By A. HAIG, M.D., F.R.C.P. Fourth Edition. 2s.

### MIDWIFERY.

**A Short Practice of Midwifery for Nurses.** By HENRY JELLETT, M.D. (Dub Univ.), F.R.C.P.I., Ex-Assistant Master, Rotunda Hospital, Dublin, Extra Examiner in Midwifery and Gynæcology, Royal College of Physicians of Ireland, Extern Examiner in Midwifery, Royal University of Ireland. With 67 Illustrations . . . . 6s.

### MONTHLY NURSING.

**A Short Manual for Monthly Nurses.** By C. J. CULLINGWORTH, M.D., F.R.C.P., Obstetric Physician to St. Thomas's Hospital. Revised with the assistance of M. A. ATKINSON, Matron of the General Lying-in Hospital, London. Fifth Edition. 1s. 6d.

### HEALTH OF A WIFE.

**Chavasse's Advice to a Wife on the Management of her Own Health.** Fourteenth Edition. By FANCOURT BARNES, M.D., Consulting Physician to the British Lying-in Hospital . . . . 2s. 6d.

### HEALTH OF CHILDREN.

**Chavasse's Advice to a Mother on the Management of her Children.** Fifteenth Edition. By GEORGE CARPENTER, M.D., Physician to the Evelina Hospital for Children . . . . . 2s. 6d.

### ANTISEPTICS.

**Lessons in Disinfection and Sterilisation**; an Elementary Course of Bacteriology, with a Scheme of Practical Experiments. By F. W. ANDREWES, M.D., F.R.C.P., Lecturer on Pathology at St. Bartholomew's Hospital. With Illustrations . 3s. net.
[*Just Published.*

### SURGERY.

**A Manual of Minor Surgery and Bandaging.** By CHRISTOPHER HEATH, F.R.C.S., and BILTON POLLARD, F.R.C.S., Surgeon to University College Hospital. Twelfth Edition. With 195 Illustrations. 6s. 6d.

### HYGIENE.

**The Elements of Health:** An Introduction to the Study of Hygiene. By LOUIS C. PARKES, M.D., D.P.H.Lond., Lecturer on Public Health at St. George's Hospital. 3s. 6d.

### DICTIONARY.

**A Short Dictionary of Medical Terms.** By R. G. MAYNE, M.D., LL.D. . 2s. 6d.

7 GREAT MARLBOROUGH STREET, LONDON.

